# Is *Toxoplasma gondii* a Trigger of Bipolar Disorder?

**DOI:** 10.3390/pathogens6010003

**Published:** 2017-01-10

**Authors:** Claudia Del Grande, Luca Galli, Elisa Schiavi, Liliana Dell’Osso, Fabrizio Bruschi

**Affiliations:** 1 Department of Clinical and Experimental Medicine, Section of Psychiatry, University of Pisa, Via Roma 67, 56127 Pisa, Italy; claudia_83@virgilio.it (C.D.G.); el.schiavi@gmail.com (E.S.); liliana.dellosso@med.unipi.it (L.D.O.); 2 Department of Translational Research and New Technologies in Medicine and Surgery, University of Pisa, Via Roma 55, 56126 Pisa, Italy; luca.galli18@virgilio.it

**Keywords:** *Toxoplasma gondii*, bipolar disorder, immunity, pro-inflammatory cytokines, dopaminergic pathways, antipsychotics and mood stabilizers

## Abstract

*Toxoplasma gondii*, a ubiquitous intracellular parasite, has a strong tropism for the brain tissue, where it forms intracellular cysts within the neurons and glial cells, establishing a chronic infection. Although latent toxoplasmosis is generally assumed to be asymptomatic in immunocompetent individuals, it is now clear that it can induce behavioral manipulations in mice and infected humans. Moreover, a strong relation has emerged in recent years between toxoplasmosis and psychiatric disorders. The link between *T. gondii* and schizophrenia has been the most widely documented; however, a significant association with bipolar disorder (BD) and suicidal/aggressive behaviors has also been detected. *T. gondii* may play a role in the etiopathogenesis of psychiatric disorders affecting neurotransmitters, especially dopamine, that are implicated in the emergence of psychosis and behavioral *Toxoplasma*-induced abnormalities, and inducing brain inflammation by the direct stimulation of inflammatory cytokines in the central nervous system. Besides this, there is increasing evidence for a prominent role of immune dysregulation in psychosis and BD. The aim of this review is to describe recent evidence suggesting a link between *Toxoplasma gondii* and BD, focusing on the interaction between immune responses and this infectious agent in the etiopathogenesis of psychiatric symptoms.

## 1. Introduction

Bipolar disorder (BD), a chronic and recurrent psychiatric illness, is one of the major causes of disability and mortality worldwide [1,2,3]. The etiology of BD is multifactorial, resulting from a complex interaction between genetic heritability and environmental risk factors [4,5]. Among these, infectious insults have been identified, and immune dysfunction seems to play a major role in the pathophysiology of this disorder, as widely documented by recent literature [6,7,8,9,10,11,12]. A chronic, mild inflammatory status in the periphery and in the brain was observed in BD patients (e.g., specific patterns of cytokines during manic/depressive episodes), and the dysregulation of inflammatory processes may also account for the medical comorbidities (atherosclerosis, hypertension, diabetes and obesity), as well as for auto-immune disorders (multiple sclerosis, thyrotoxicosis, ulcerative colitis, psoriasis and rheumatoid arthritis) that commonly occur in association with BD [11,12].

Although further evidence is needed to clarify the pathogenetic mechanisms underpinning BD, immunological abnormalities may help to explain the cyclic and recurrent course of the disease characterized by marked fluctuations in mood; moreover, they could allow one to identify novel promising neuroimmunological targets for the treatment of BD [13,14].

**Figure 1:**
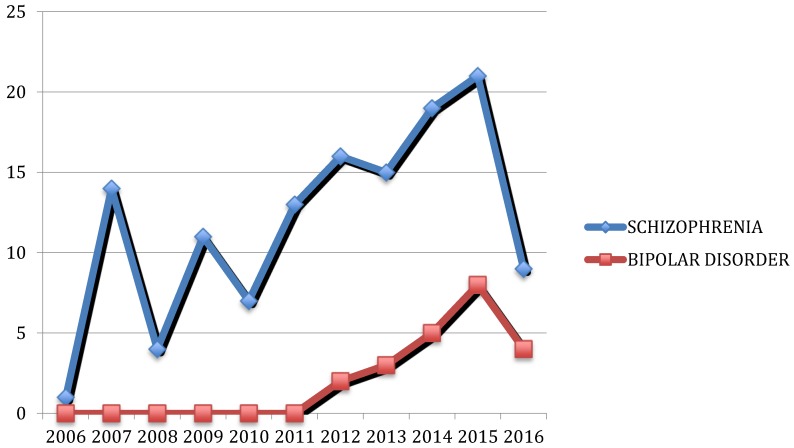
Publications available in PubMed in the last 10 years focusing on the relationship between schizophrenia or bipolar disorder and toxoplasmosis. Search terms were “*Toxoplasma gondii*”, “toxoplasmosis”, “neuropsychiatric disorders”, “bipolar disorder”, “schizophrenia”, “mania”, “psychotic symptoms”. We only included papers assessing schizophrenia and bipolar disorder.

The implication of infectious agents, particularly *Toxoplasma gondii* (*T. gondii*), in the development of major psychosis has recently gained increasing attention [15,16,17,18,19,20,21]. In the last decade, the number of publications dealing with the relationship between toxoplasmosis and schizophrenia or BD has dramatically increased, as shown in Figure 1. However, the role of infectious agents remains controversial, and to date, no clear cause-effect etiopathogenetic relation can be established.

*T. gondii* is an intracellular protozoan parasite, which infects around one-third of humans worldwide, with prevalence rates that can reach up to 95% in the elderly population in some geographical areas [22,23,24]. In humans, *T. gondii* is commonly acquired by the oral ingestion of tissue cysts containing bradyzoites, through the consumption of undercooked meat infected with *T. gondii*; however, it can also be acquired via ingestion of the parasite’s oocysts, which can spread from the feces of infected cats [25,26] or through contaminated water [27]. This parasite has a particular tropism for muscle and brain tissue, where it remains localized in the form of cysts throughout life, establishing a chronic infection. The clinical presentation of infection may be heterogeneous, depending to a great extent on the immune status of the host, which is determined by both genetic and environmental factors [28]; it can range from an asymptomatic or negligible symptomatic form mostly observed in immune-competent individuals to severe forms presenting with encephalitis or multi-organ involvement, more frequently in immunocompromised hosts [22,26]. *T. gondii* has been shown to be a potent disrupter of fetal neurodevelopment, while congenital infection, occurring when a pregnant woman has a primary infection and transmits *T. gondii* to the fetus, has been associated with uveitis, chorioretinitis, encephalitis, epilepsy and psychomotor or mental retardation [29,30].

This review aims to describe the relationship that has emerged in recent years between toxoplasmosis and psychiatric disorders, focusing mainly on BD. The possible etiopathogenetic mechanisms underpinning this association were analyzed, emphasizing how the inflammatory damage induced in the brain tissue by the parasite might contribute to the emergence of clinical manifestations, as well as the progression of BD.

## 2. Relationship between *Toxoplasma gondii* and Bipolar Disorder

The first observations of an involvement of infectious agents, such as viruses, spirochetes and protozoa, in the pathogenesis of psychosis were described more than a century ago, as reviewed by Yolken and Torrey [15]. Infectious agents can cause psychosis both directly, by affecting neurons and brain structures, and indirectly, by the activation of a microbe-specific immune response and the subsequent release of neurotoxic factors [31,32].

A number of epidemiologic studies has recently highlighted the prominent role of *T. gondii* in the pathogenesis of several psychiatric disorders. Although latent toxoplasmosis was generally assumed to be relatively harmless in immune-competent individuals, there is now evidence that it may cause personality and behavioral changes in chronically-infected hosts, such as a reduction in reaction time and psychomotor performance, reduced intelligence quotient and aggression/impulsivity traits [33,34,35,36,37,38,39,40]. These observations are supported by prior animal models showing behavioral changes in *T. gondii-*infected mice, such as conversion from aversion to attraction to cat odor, motor coordination and sensory deficit and reduced exploratory activity [41,42,43,44]. It has been suggested that these behavioral changes may be caused by manipulation activity of the parasite aiming to increase the probability of its transmission from an intermediate to a definitive host [45].

*T. gondii* infection has also been associated with major psychosis [15,19,21,46,47] and with self-directed violence and suicidal behaviors [48,49,50,51,52]. In this context, the link between toxoplasmosis and schizophrenia has been the most widely investigated, with 42 studies showing higher rates of anti-*T. gondii* IgG antibodies in patients with schizophrenia as compared to psychiatrically healthy controls [16,53]. Nevertheless, an association between *T. gondii* exposure and the risk of BD has increasingly emerged in recent years [20,21,54,55,56].

A first study using a toxoplasmin intradermal test showed that high grade positivity was observed among patients with manic-depressive illness [57], and a more recent Ethiopian case-control study revealed a significantly higher seroprevalence of *T. gondii* infection in BD patients compared to healthy controls [58]. Some authors [36] also found that in a sample including 896 psychiatric inpatients with the primary diagnoses of schizophrenia, major depression, schizoaffective disorder or BD and 214 psychiatrically unaffected controls, the additional diagnosis of a personality disorder was significantly associated with *T. gondii* infection among all psychiatric inpatients. These data support that *T. gondii* infection can affect human behavior and personality traits.

In a cross-sectional survey conducted on a sample of 7440 individuals of 15–39 years old from the Third National Health and Nutrition Examination Survey, the association between *T. gondii* exposure and several well-defined mood disorders was examined [56]. Results of this study showed a significant relationship between *Toxoplasma* seropositivity and a subtype of BD type I in which both manic and depressive features were reported. Respondents with a prior *T. gondii* exposure were approximately 2.3-fold more likely to have a history of BD type I with manic and depressive symptoms than respondents who tested negative for *T. gondii* antibody. The authors hypothesized that the infection with the parasite may precipitate or accelerate depressive symptoms in patients with this subtype of BD. Conversely, no statistically-significant association was found between *T. gondii* seroprevalence and a history of major depression or dysthymia, according to observations from prior smaller studies [48,59,60]. It has been hypothesized that the positive association found between a subtype of BD type I and toxoplasmosis could be due to behavioral factors, probably more common among individuals with this mood disorder, exposing the patients to a greater risk of infection with the parasite. On the contrary, it is possible that major depressive disorders decrease the risk of behaviors related to *T. gondii* exposure [58]. Another case-control seroprevalence study investigated the potential association between *T. gondii* exposure and BD comparing the prevalence of anti-*T. gondii* IgG/IgM antibodies in a sample of 110 BD and 106 healthy controls all living in France, a country of high seroprevalence of toxoplasmosis [20]. Results of this study showed that both the seropositivity and the *T. gondii* antibody levels were significantly higher in BD patients as compared to controls; moreover, the seropositive participants had a 3.6-fold increased odds of having BD as compared to the seronegative group (*p* < 0.0001), with an odds ratio (OR) of 2.40 (*p* = 0.026) after adjustment for age, ethnic origin, foreign birth and current smoking. This OR is quite similar to that previously observed in samples of schizophrenic patients (OR = 2.70–2.73) [18,55]. Dickerson et al. [61] examined the levels of serum IgG and IgM antibodies to *T. gondii* and CMV antibodies in 57 patients hospitalized with a manic episode and in 743 individuals in other psychiatric groups belonging to bipolar or schizophrenic disorders compared with those in 314 non-psychiatric controls. Patients in the mania group were assessed up to three time points, and anti-*T. gondii* antibody levels were compared over time in this group. Results of this study showed that manic patients had a significantly elevated level of IgM antibodies to *T. gondii* at baseline as compared to the control group without a psychiatric diagnosis. Elevated anti-*T. gondii* IgM antibodies were not found in patients with the other psychiatric diagnoses, while the group of patients with a recent onset of psychosis alone had significantly elevated levels of IgG antibodies to *T. gondii*. Within the mania group, a significant difference between the prevalence of increased levels of *T. gondii* antibodies at the baseline and the follow-up time-point was detected. None of the groups had higher levels of IgG or IgM antibodies to CMV. Since elevated levels of IgM antibodies to *T. gondii* following reactivation or re-infection have been reported, Dickerson et al. [61] hypothesized that individuals with elevated IgM had undergone infection with or reaction to *T. gondii* around the time of their hospitalization for a manic episode, according to the fact the IgM antibody levels decreased over time. It is also possible that patients in the manic group display an aberrant production of antibody, perhaps related to aberrant class switching. Finally, a recent study conducted on a sample of 70 military women veterans analyzed for *T. gondii* IgG titer described biobehavioral relationships between chronic *T. gondii* infection, depression and dysphoric moods [62]. In contrast to the above studies, it is worth mentioning a recent study performed to evaluate the *Toxoplasma* seroprevalence of patients with BD I compared to healthy controls in Iran, a country with a very high prevalence of toxoplasmosis [63]: the authors showed no significant difference between BD I patients and healthy subjects in IgG antibodies (31.62% and 26.5%, respectively). In Table 1, we summarize the published sero-epidemiological studies investigating the association between *T. gondii* infection and BD.

We recently described the case of a 20-year-old Brazilian female affected by recurrent ocular toxoplasmic uveitis and BD with psychotic features. In this patient, a consistent relationship between ocular manifestations of *T. gondii* infection and the onset and recurrences of psychiatric symptoms was detected. Infection reactivation was directly documented through molecular analyses (nested-PCR), showing the presence of circulating DNA parasite at the time of occurrence of psychiatric symptoms and the high level of *T. gondii*-specific IgG [64].

Prenatal exposure to viruses or parasite with tropism for the central nervous system (CNS) is known to be a risk factor for later development of psychotic disorders [65,66]. Several studies showed that increased levels of maternal antibodies to *T. gondii* are associated with a higher risk of schizophrenia spectrum disorders in adult offspring [65,67]. Contrary to this, no significant association was found between prenatal exposure to *T. gondii* and the risk of BD in adult offspring, suggesting that congenital *T. gondii* infection could be a risk factor only for the development of schizophrenia and related psychosis, but not for BD [65,68,69]. Only one study, investigating whether certain *Toxoplasma* genotypes may be differentially associated with the risk of psychosis, found that the offspring of mothers with a serological pattern consistent with *Toxoplasma* genotype I infection were at significantly increased risk for the development of psychosis as compared with the matched unaffected control mothers, and the risk was statistically significant only for affective psychosis, including schizoaffective disorder-bipolar type, BD with psychotic features and major depressive disorder with psychosis. Conversely, no significant association between serological evidence of infection with other *Toxoplasma* genotype and offspring psychoses was detected [70]. The studies that investigated the relations between perinatal toxoplasmosis and BD are summarized in Table 2.

In addition, *T. gondii* has been linked to suicidal and aggressive behavior in patients with recurrent mood disorders and in younger patients with schizophrenia [48,50,71]. In a survey conducted in 20 European countries, a significant association between *T. gondii* seropositivity and national suicide rates was observed [72]. This would indicate that latent toxoplasmosis, along with other established suicide factors, might contribute to increasing the rates of suicide among BD patients. This hypothesis is corroborated by the observation that *T. gondii*-infected women have an increased risk of self-directed violence and suicide [49,51]. Furthermore, early reports found a significant association between seropositivity and T*. gondii*-specific IgG levels with non-fatal suicidal self-directed violence, which represents the most significant risk factor for completed suicide [48,52,73]. Given the strong relationship between suicidal and impulsive/aggressive behavior, Coccaro et al. [71] tested the hypothesis that IgG antibodies to *T. gondii* may be associated with higher aggression and impulsivity scores in a sample of 358 adult subjects with a DSM-5 diagnosis of intermittent explosive disorder (IED), non-IED psychiatric disorders (psychiatric controls) or psychiatrically healthy controls assessed with psychometric measures of aggression, impulsivity and related behaviors. Results of this study showed that *T. gondii* seropositivity status was associated with higher scores on the psychometric measures for aggression; the rate of *T. gondii* seropositivity in IED patients was significantly greater than that in healthy controls, although no significant difference was found between IED and non-IED patients in the seropositivity rate.

On the basis of available literature data, a *T. gondii* seropositivity status seems to be mostly associated with a greater prevalence of psychotic symptoms in schizophrenia patients, a greater number of depressive symptoms in BD patients and a chronic course of the psychiatric disease with higher suicidal and impulsive/aggressive behaviors [48,49,51,52,56,71,73,74]. However, the clinical characteristics and illness course of BD patients with a seropositivity to *T. gondii* in comparison with seronegative BD patients need to be more clearly defined.

## 3. The Role of *T. gondii* in the Etiopathogenesis of Psychiatric Disorders

Despite the great number of studies showing a relationship between *T. gondii* and the onset or recurrence of major psychosis, as well as human behavioral changes, it is difficult to clearly establish the role of *T. gondii* infection in the pathogenesis of psychiatric disorders. Direct and indirect etiopathogenetic mechanisms, including the permanent resident glia cell activation and the recruitment of peripheral immune cells, as well as effects on neurotransmission have been proposed.

Neuropathological studies have reported that glial cells, especially astrocytes, are selectively affected in both toxoplasmosis and schizophrenia, supporting their involvement in the emergence of *T. gondii*-related psychosis [75,76]. *T. gondii* probably migrates to the brain as early as seven days post-infection and crosses the blood-brain barrier through a “Trojan horse mechanism”. Alternatively, replication of *T. gondii* and lysis of infected endothelial cells could be required for the protozoan to cross the blood-brain barrier [77].

After reaching the CNS, the parasite can invade all nucleated cells and initiate the activation of resident microglia and astrocytes, the resident macrophages of the brain that will produce pro-inflammatory cytokines and free radicals, but also anti-inflammatory components; in murine models, it was found to infect 30% of microglial cells and 10% of neurons and astrocytes [78]. Initial signs of glia activation can be observed between seven and 10 days post-infection and are followed by the recruitment of peripheral T cells and mononuclear cells. The early activation of microglia and astrocytes correlates with the local production of chemokines and cytokines, which contributes to immune cell recruitment from the periphery. The effective control of parasite replication and disruption of the parasitophorous vacuole is dependent on cell extrinsic mechanisms, as well as cell intrinsic mechanisms, mediated by cytokine signals [79]. Within weeks or months, tachyzoites disappear, but bradyzoites remain in tissue cysts throughout the life of the host. Several in vitro studies found that the parasite can develop cysts in neurons and astrocytes [78], but a recent study showed that in the chronic phase of infection, cysts are detectable only in neurons [80].

The distribution of *T. gondii* cysts in the brain has been suggested to play an important role in the behavioral adaptive manipulation produced by the parasite in infected hosts [81]. In this regard, several studies suggest that *T. gondii*-containing cysts localize preferentially in the prefrontal cortex and amygdala, a structure involved in the regulation of fear behavior, although found throughout the brain [42,81,82]. Latent toxoplasmosis was also shown to induce dendritic retraction in basolateral amygdala accompanied by reduced corticosterone secretion, which may deal with *T. gondii*-induced behavioral changes [83]. However, other studies reported widespread parasite cyst localization in infected rats rather than a specific localization to a certain brain region and showed a considerable inter-individual variation in tissue cyst density in different brain areas [84]. These data are corroborated by neurobiological studies showing that brain cysts are formed in the whole brain (cerebral hemispheres, hippocampus, amygdala, basal ganglia, cerebellum, cerebral cortex, brain stem and olfactory bulb) [47].

A *T. gondii*-induced alteration of the structure and function of corticolimbic circuits, which are involved in the modulation of impulsivity and aggression, could be responsible for behavioral changes observed in infected animals and humans [71,85]. Specifically, persistent *T. gondii* infection in mice has been associated with neuronal tissue lesions, impaired neuronal functions, ventricular dilation and neuroinflammation, suggesting that latent toxoplasmosis might contribute to neuroinflammation or neurodegeneration in genetically susceptible hosts [86].

Besides the proven neurotropism of *T. gondii*, the link between this parasite and psychiatric disorders could be also explained by its ability to influence neurotransmitter pathways. Indeed, *T. gondii* has been shown to increase dopamine (DA) levels, as well as to modulate serotonin, gamma-aminobutyric acid and glutamate signaling, all of which are implicated in aggressive behavior and in the pathogenesis of psychotic syndromes in human studies [16,87,88,89,90].

An increased metabolism of DA due to parasite infection has been observed especially in the limbic region, known to be altered in BD [91]. Gaskell et al. [92] have demonstrated that the parasite genome includes two genes encoding enzymes with tyrosine hydroxylase (TH) activity, and one of these genes is induced during the production of *T. gondii* bradyzoites. TH is the rate-limiting enzyme for DA synthesis in the brain, catalyzing the conversion of L-tyrosine to L-dihydroxyphenylalanine. In vitro studies showed that DA itself may play a role in the production/proliferation, chemoattraction, infection efficiency and stage conversion of *T. gondii* in the brain [93]. Prandovszky et al. [91], using both mammalian dopaminergic cells infected with *T. gondii* and brain sections of infected mice, showed a significant increase in DA metabolism in neural cells, with a direct correlation between the number of infected cells and the quantity of DA released. In this study, TH was also found in intracellular tissue cysts in brain tissue using antibodies specific for the parasite-encoded tyrosine hydroxylase. The *T. gondii*–induced release of DA has been proposed as a relevant neurochemical mechanism for parasite-induced psycho-behavioral abnormalities in *Toxoplasma*-infected rodents and possibly in humans [45,91,94].

Chronic *T. gondii* infection is also responsible for immunological alterations, such as a higher production of cytokines and other inflammatory mediators, in the brain of infected hosts [90]. In different stages of infection, the parasite induces the production of several cytokines, such as interferon (IFN)-γ, tumor necrosis factor (TNF)-α, interleukin (IL-)1, IL-1β, IL-2, IL-4, IL-6, IL-10, IL-12 IL-17, IL-23 and granulocyte macrophage colony-stimulating factor (GM-CSF), which are variably expressed by microglia cells, astrocytes and infiltrating CD4+ and CD8+ T cells [47]. These inflammatory modifications may have the downstream effect of modulating neurotransmission, leading to the occurrence of psychiatric symptoms and aggressive behavior [90,95,96]. Further, an association between increased peripheral levels of pro-inflammatory cytokines, such as IL-6 and TNF, and elevated cerebrospinal fluid levels of IL-6, in response to toxoplasmosis, has recently been associated with self-directed violence and suicide attempts [71,97,98].

A pro-inflammatory state involving both the innate and the adaptive immune response is well documented in depressive and manic episodes of BD, and a great amount of evidence indicates that inflammatory processes play a major role in the pathophysiology of this psychiatric disorder [9,11,99,100]. Human and animal studies have in fact demonstrated that mood symptoms can be induced by the activation of inflammatory reactions [101,102,103,104]. Moreover, many studies have documented specific patterns of increased levels of cytokines in different phases of BD: namely, the pro-inflammatory cytokines increased during mania seem to be IL-2, IL-6, IL-8 and INF-γ, whereas only IL-6 is increased during depressive episodes, with an imbalance between IL-6 levels and those of the anti-inflammatory cytokine IL-10 [105]. Elevated peripheral levels of TNF-α have been reported in BD patients during manic and depressive episodes [8], and cerebral spinal fluid (CSF) IL-1β levels were particularly increased in those patients with a history of BD with prominent psychotic symptoms [106]. More recently, the levels of soluble TNF receptor type 1 were found to be significantly lower in BD type II patients than in patients with BD type I, as well as in depressed BD patients compared to patients with manic/hypomanic and euthymic states [107]. Successful treatment with mood stabilizers leading to a euthymic state has been associated with a significant reduction of pro-inflammatory cytokines in BD subjects, with IL-6 levels returning to baseline after anti-manic and mood-stabilizing treatments [108,109,110]. Overall, these data suggest that manic phases and, to a lesser degree, depressive phases of BD are associated with a persistent and chronic low-grade pro-inflammatory state [9,11].

A recent study [55] observed a significant correlation between cognitive deterioration, IL-6 levels and *T. gondii* seropositivity in BD patients. According to this, growing data suggest that the “pro-inflammatory cytokines state” could be one of the mechanisms involved in cognitive deterioration in BD [111,112]. Long-term inflammation observed in *T. gondii*-infected BD patients may progressively impair cognitive functions through the disruption of neurogenesis processes, and IL-6 serum levels could be a useful and predictive immunological marker of cognitive decline in BD, as well as a valuable tool for designing personalized treatment.

*T. gondii*-induced cytokines, particularly IL-6, IL-1β and TNF-α, could indirectly affect neuronal function by the influence exerted on the hypothalamic-pituitary-adrenal axis (HPA), which is known to be linked to behavioral changes and to increased glucocorticoid levels [47]. These hormones act on the HPA axis with a negative feed-back mechanism reducing neuroplasticity and cellular resistance. This action may lead to an imbalance between glucocorticoids, mineralocorticoids and their high-density receptors, as well as to neuronal injury. In addition, the higher activity of the HPA axis induces the activation of macrophages, dendritic cells and T cells and the production of pro-inflammatory cytokines, with a positive feed-back mechanism. Glucocorticoids also increase the levels of tryptophan 2,3-dioxygenase (TDO) in the liver and the level of indoleamine 2,3-dioxygenases (IDO) in immune cells. IDO and TDO catalyze the opening of the tryptophan (TRP) indole ring, the first step for the synthesis of kynurenic acid. In the brain, kynurenine (a precursor of kynurenic acid) synthesis takes place mainly in astrocytes, which release newly-produced kynurenic acid into the extracellular environment, affecting the surrounding neurons. Immune response could interfere with the growth and survival of the parasite through the degradation of TRP to *N*-formylkynurenine [113].

The role of the kynurenine pathway is well defined in schizophrenic patients [114]; the first description of kynurenine upregulation in BD was published by Miller et al. [115], which observed increased levels of both kynurenine and kynurenic acid in the anterior cingulate in BD patients with a history of psychotic symptoms in post-mortem studies. A few years later, CSF studies confirmed an increase in kynurenic acid notably restricted to BD patients with a history of psychosis [116]. In line with data from the CSF and post-mortem studies, cultured human dermal fibroblasts obtained from patients with BD or schizophrenia are shown to release significantly more kynurenic acid than cells obtained from controls [117].

Finally, experimental studies in rats showed *T. gondii* infection to increase the expression of genes involved in the production of testosterone, a steroid that is known to reduce fear and enhance sexual attractiveness in males [71,118]. Additionally, there is evidence that *Toxoplasma*-infected men have a higher concentration of testosterone than *Toxoplasma*-free controls, while the opposite direction of testosterone shift has been detected in *Toxoplasma*-infected women. These findings could explain the observed gender specificity of behavioral shifts in *Toxoplasma*-infected subjects [119].

## 4. The Association between Autoimmune/Inflammatory Dysregulation, Infectious Agents and Risk of Bipolar Disorder

Autoimmune processes, as well as activated peripheral and central inflammatory responses, have been described in both schizophrenia and BD, supporting the view that dysregulation of the immune system represents an important vulnerability factor for psychosis [32,120,121,122].

Animal models of psychotic conditions have suggested that infections and other environmental stressors during gestation/early life can activate microglia cells, which have been increasingly implicated in neurodevelopmental processes, thereby disrupting brain development and setting the stage for vulnerability for later psychotic disorder [18,32,123]. A second environmental, infectious or stress-related trigger in later life could further activate microglia and mast cell release of pro-inflammatory cytokines, thus contributing to brain inflammation and psychosis [32,124]. Since *T. gondii* is known to be a potent disrupter of fetal neurodevelopment, the parasite-induced microglia activation and the subsequent induction of an inflammatory environment in the brain could be the mechanisms linking toxoplasmosis to the higher vulnerability to subsequent psychosis.

Several epidemiologic studies have demonstrated the high co-occurrence of autoimmune diseases, chronic inflammatory conditions and psychotic or mood disorders [121,125], leading some authors to hypothesize a common underlying immune abnormality for both autoimmunity and mental illness, at least in specific subgroups of psychotic patients [32]. According to this hypothesis, autoimmune diseases and psychotic disorders, mainly affective ones, exhibit common clinical characteristics, which is high familiarity, progression from prodromal subclinical to clinical disease and a cyclical exacerbation-remission pattern [32].

A nationwide, population-based, prospective cohort study [126] showed that any history of hospitalization for infection or autoimmune disease increased the risk of a subsequent mood disorder diagnosis by 62% and 45%, respectively, and interestingly, the two risk factors interacted synergistically. In a genome-wide study comparing schizophrenia cases, BD cases, parents of cases and screened controls, Avramopoulos and collaborators [127] tested the hypothesis that environmental exposure to *T. gondii*, herpes simplex virus (HSV)-1, CMV, human herpes virus 6 and the food antigen gliadin may increase the risk for schizophrenia and BD and that the increased risk may be dependent on genetic variants. This study found that patients had higher seropositivity rates than controls for any of the serological analysis and higher C-reactive protein levels in both BD and schizophrenia, according to existing literature data [128,129]. Finally, another recent gene-environment interaction study investigated the possible effect of interactions between serologically documented exposure to *T. gondii*, CMV, HSV-1 or HSV-2 and polymorphisms of *TLR2*, *TLR4* and *NOD2* genes, which encode for pivotal pattern-recognition receptors, in a sample of 138 BD patients [130]. The genetic associations between *TLR2*, *TLR4* and *NOD2* polymorphisms with susceptibility to infections, on the one hand, and BD, on the other, have been previously reported in the literature [131,132,133,134,135,136]. Oliveira et al. [130], besides confirming the association between BD and *T. gondii*, first described a trend for an interaction between a *TLR2* polymorphism and *T. gondii* seropositivity in conferring BD risk, suggesting that exposure to infection may modulate the immunogenetic background on BD.

Although preliminary, these findings highlight the close link between autoimmune/inflammatory processes, infections and the risk of psychosis and mood disorders, supporting the immunological hypothesis in the pathogenesis of at least specific subgroups of schizophrenic and BD patients.

## 5. Anti-Toxoplasmic Activity of Antipsychotics and Mood Stabilizers

It is noteworthy that some antipsychotic medications, commonly used for the treatment of BD and schizophrenia, have been demonstrated in vitro to possess antiprotozoal properties.

In a study investigating the levels of antibodies to infectious agents in the serum and CSF of individuals with recent onset schizophrenia in comparison with those of controls without psychiatric disease, untreated individuals with recent onset schizophrenia were found to have significantly increased levels of serum and CSF IgG antibodies to *T. gondii* as compared to controls. A significant reduction of *T. gondii* antibodies was found in those patients undergoing current drug treatment, suggesting that anti-psychotic medications may affect *T. gondii* replication [137]. However, conflicting results have been reported on the anti-toxoplasmic activity of psychotropic drugs. Jones-Brando and collaborators [138] investigated the relative ability of eight antipsychotics and four mood-stabilizing medications to inhibit cell invasion and/or replication of *T. gondii* tachyzoites in a cell culture system using human fibroblasts treated with test compounds and then exposed to *Toxoplasma*, in comparison with the antiprotozoal activity of trimethoprim, a folate antagonist commonly used for the treatment of toxoplasmosis. Among the tested medications, the antipsychotic haloperidol and the mood stabilizer valproic acid and its salt, sodium valproate, showed the most robust inhibitory activity, followed by 9-OH-risperidone, the principal metabolite of risperidone, and fluphenazine. It is of interest that this is the first study reporting an in vitro activity against T. *gondii* of a mood stabilizer, with therapeutic indices of valproic acid and sodium valproate equivalent to that of trimethoprim. Moreover, valproic acid was found to inhibit the parasite at a lower concentration than that found in the CSF and blood of individuals being treated with this mood stabilizer and displayed synergistic inhibitory activity with both haloperidol and trimethoprim. The other antipsychotics, such as chlorpromazine, had a modest therapeutic index, while carbamazepine was demonstrated to have only a small inhibitory effect, and anti-*Toxoplasma* activity was not found for lithium salts. In light of these findings, Webster et al. [139] hypothesized that the antipsychotic and mood-stabilizing activity of some medications may be achieved, or at least augmented, through their inhibition of *T. gondii* replication and invasion in infected individuals. In particular, these authors predicted that haloperidol and valproic acid would be at least as effective in preventing or alleviating *T. gondii*-induced cognitive and behavioral alterations as the standard anti-*T. gondii* chemotherapeutics pyrimethamine with dapsone. Results of this study showed that treatment of infected rats with, in order of decreasing efficacy, haloperidol, dapsone and valproic acid reduced the predatory risk behavioral traits observed in untreated rats. Since *T. gondii* tachyzoites require calcium in order to invade host cells, the anti-toxoplasmic activity of these drugs was supposed to be partly related to their calcium inhibitory properties [138,140,141]. More recently, Fond et al. [142] found that fluphenazine has a high inhibitory activity of parasite replication in vitro, in agreement with previous findings of Goodwin et al. [143], but also that zuclopenthixol, another first-generation antipsychotic, has a high activity against the parasite growth, therefore invalidating the previous hypothesis of a phenothiazine-specific class effect [143]. These authors also confirmed that haloperidol has no anti-toxoplasmic activity and performed a preliminary assay on several antipsychotics that have never been reported before, such as amisulpride, which showed no anti-toxoplasmic effect, and levomepromazine, loxapine and cyamemazine, which showed intermediate anti-toxoplasmic activity. Contrary to previous findings [138], in this study, valproate has no effect on parasite replication. Valproic acid was also found to be not effective in vivo in preventing acute toxoplasmosis in mice inoculated with *T. gondii* tachyzoites; additionally, no activity against tissue cysts was observed in chronically *T. gondii*-infected valproic acid-treated mice [144].

In a recent cross-sectional retrospective study, Fond et al. [145] evaluated the effect of the administration of a psychotropic drug having known in vitro anti-toxoplasmic activity (TATA+) on clinical outcome in a population of bipolar or schizophrenic/schizoaffective patients with a seropositivity to *T. gondii*, compared to patients receiving a treatment without anti-toxoplasmic activity (TATA−). Cyamemazine, fluphenazine, haloperidol, levomepromazine, loxapine, paliperidone, risperidone, thioridazine, zuclopenthixol and valproate were considered as TATA (“TATA+”), while amisulpride, aripiprazole, carbamazepine, clozapine, lamotrigine, lithium carbonate, olanzapine, quetiapine and tiapride were considered as having no or negligible anti-toxoplasmic activity (“TATA−”). The authors observed that a current TATA+ treatment was associated with lower lifetime number of depressive episodes (*p* = 0.048), but not with a lower number of manic or psychotic episodes. A significant difference was not found in BD toxo-negative patients, nor in schizophrenic toxo-positive or toxo-negative patients regarding lifetime and current mood or psychotic symptomatology. Results of this study suggest that toxoplasmic serological status may be a biomarker of interest for the prevention of bipolar depression through the administration of TATA+ drugs; moreover, they may partly explain discrepancies detected in the effectiveness of valproic acid, the mood stabilizer with the highest demonstrated anti-toxoplasmic activity in bipolar depression across studies [146]. Since depressive symptoms and episodes dominate the long-term course of BD and are associated with high levels of disability and an increased suicide risk [147], findings from the study of Fond et al. [145], if confirmed in further prospective randomized controlled trials, may have important implications for the management of *Toxoplasma*-seropositive BD patients.

## 6. Conclusions

In recent studies, infectious agents emerged as a group of well-defined environmental risk factors in psychiatric disorders, particularly schizophrenia and BD. The association between *T. gondii* seropositivity and schizophrenia/schizoaffective disorder is one of the most studied links between one pathogen and psychiatric disorder. However, growing emerging evidence has also documented an association between latent toxoplasmosis and the risk of BD and suicidal/aggressive behaviors. *T. gondii* infection, as previously suggested for schizophrenia [113], may contribute to the onset and the progressive course of the disease interacting with genetic hereditary predisposing factors, as well as affecting neurotransmitter systems and immune responses, which have recently been shown to be closely linked to the pathogenesis of BD and its medical comorbidities.

Despite the fact that evidence supporting a relationship between toxoplasmosis and BD has been presented in this review, this area remains controversial without cause-effect studies, and it is difficult, at present, to establish the direction of causality between *T. gondii* and BD; in fact, if the parasite could directly trigger a BD recurrence [9,138], the possibility that the modifications of the immune response, caused by a recrudescence of BD, can induce a reactivation of toxoplasmosis has to be also considered, as previously hypothesized for schizophrenia [15]. Certainly, further research is needed to investigate the role of *T. gondii* in larger samples of BD patients also including BD type II diagnosis and different stages of illness. Finally, it seems to be of importance to consider that the identification, prevention and treatment of infections like toxoplasmosis contributing to the etiopathogenesis of schizophrenia and BD could have a significant impact on the disease course. For this reason, every attempt to prevent toxoplasmosis should be encouraged, waiting for a reliable vaccine in humans.

## Figures and Tables

**Table 1:** Sero-epidemiological studies investigating the association between *T. gondii* infection and bipolar disorder.

Authors (Year), Country	Study Design	Toxo Determination	Sample Size	Description of Association	Limitations
Delgado Garcia, Rodriguez Perdomo (1980) [57], Cuba	Case-control study	Toxoplasmin intradermal test	270 individuals (50 pts with manic-depressive psychosis, 120 neurotics, 100 HC)	The highest percentage of reactors was found among pts with manic-depressive psychosis (66.0%); the intensity of reaction was also higher among these pts.	Small sample size
Hinze-Selch et al. (2010) [36], Germany	Cross-sectional study	IgG levels	1110 (270 SCH, 465 MD, 67 schizoaffective disorder, 87 BD, 214 HC)	Additional diagnosis of a personality disorder is significantly associated with *T. gondii* infection among all psychiatric inpatients.	Small sample size of BD pts
Tedla et al. (2011) [58], Ethiopia	Case-control study	IgG levels	495 (214 SCH, 171 BD, 71 HC)	Higher *T. gondii* seroprevalence in individuals with SCH (OR = 4.7) and BD (OR = 3).	No IgM determination
Pearce et al. (2012) [56], USA	Cross-sectional study	IgG levels	7440 (individuals 15–39 years old from the Third National Health and Nutrition Examination Survey)	Significant relationship between *Toxoplasma* seropositivity and lifetime history of a BD I subtype with manic and depressive episodes (adjusted OR = 2.4, *p* < 0.05). No statistically-significant association between *T. gondii* seroprevalence and a history of MD or dysthymia.	Small number of respondents with both BD I and positive serology for *T. gondii*; no IgM determination
Hamdani et al. (2013) [20], France	Case-control study	IgM/IgG levels	216 (110 BD, 106 HC)	BD pts had significantly higher seropositivity to *T. gondii* and higher anti-*T. gondii* IgG titers as compared to HC (76.9% vs. 48.2%, *p* = 0.00045; 3.06 ± 1.07 vs. 2.07 ± 1.9, *p* = 0.002). IgM antibodies detection was negative for the whole sample. Seropositive participants had a 3.6-fold odds risk of BD as compared to seronegative group (*p* < 0.0001) (adjusted OR = 2.40, *p* = 0.026).	Small sample size
Dickerson et al. (2014) [61], USA	Cross-sectional study	IgM/IgG levels assessed up to three time points	1114 (57 individual with mania, 743 other psychiatric groups, 314 non-psychiatric controls)	Significantly elevated levels of anti-*T. gondii* IgM in pts with mania as compared to non-psychiatric controls (OR = 2.33, *p* < 0.04 at hospital admission; OR = 2.32, *p* < 0.02 at study entry during the hospital stay). Significant difference between the prevalences of increased levels of anti-*T. gondii* IgM at the baseline and the follow-up time-point (*t* = 2.97, *p* < 0.003). Significantly elevated levels of IgG antibodies to *T. gondii* (OR = 2.42, *p* < 0.04) in the recent onset psychosis group alone.	Time of *T. gondii* infection is unknown; clinical assessments only during the hospital stay; lack of measurement of symptom scores at hospital admission
Khademvatan et al. (2013) [63], Iran	Cross-sectional study	IgM/IgG levels	317 (117 BD I; 200 HC)	No significant difference in prevalence of anti-*T. gondii* IgG antibodies between BD I pts and HC (31.62% vs. 26.5%, *p* = 0.3). No significant difference between IgM levels in the two groups.	No BD II pts
Duffy et al. (2015) [62], USA	Cross-sectional study	IgM/IgG levels	70 (veteran women)	Positive relationships between *T. gondii* and depressive symptoms, anger, confusion and overall mood disturbance.	Small sample size

SCH: schizophrenia; MD: major depression; pts: patients; BD: bipolar disorder; HC: healthy controls.

**Table 2:** Studies on the relations between perinatal infection with *T. gondii* and bipolar disorder.

Authors (Year), Country	Study Design	Toxo Determination	Sample Size	Description of Association	Limitations
Xiao et al. (2009) [70], USA	Case-control study	IgG levels and serotyping	837 (219 sera from pregnant women whose children developed SCH and affective psychotic illnesses in adult life and 618 matched unaffected control mothers)	No association between maternal antibodies to *T. gondii* and risk of psychoses in the offspring. Only the offspring of mothers with a serological pattern consistent with *Toxoplasma* type I infection are at significantly increased risk for the development of psychosis (*p* = 0.03). The elevated risk for subsequent psychoses associated with maternal infection with the type I strain is statistically significant for patients with affective psychosis (*p* = 0.005), but not for those with schizophrenic and other non-affective psychosis.	Moderate sensitivity of serotyping; unknown indicators of the timing of maternal infection
Mortensen et al. (2011) [68], Denmark	Case-control study	IgG levels (neonatal dried blood spots)	154 (127 BD pts and 127 matched controls)	No association between marker of *T. gondii* prenatal infection and risk of BD.	Small study samples; absence of information about specific serotypes of *Toxoplasma;* unknown indicators of the timing of maternal infection
Freedman et al. (2016) [69], USA	Case-control study	IgG levels (maternal sera)	255 (85 BD cases and 170 comparison subjects)	Maternal *T. gondii* IgG is not associated with the risk of BD in offspring.	Small sample size; absence of information about specific serotypes of *Toxoplasma*; unable to determine when the infection occurred in relation to the pregnancy or birth

SCH: schizophrenia; pts: patients; BD: bipolar disorder.
